# Association of Mitochondrial DNA Haplogroups with Exceptional Longevity in a Chinese Population

**DOI:** 10.1371/journal.pone.0006423

**Published:** 2009-07-29

**Authors:** Xiao-yun Cai, Xiao-feng Wang, Shi-lin Li, Ji Qian, De-gui Qian, Fei Chen, Ya-jun Yang, Zi-yu Yuan, Jun Xu, Yidong Bai, Shun-zhang Yu, Li Jin

**Affiliations:** 1 State Key Laboratory of Genetic Engineering and MOE Key Laboratory of Contemporary Anthropology, School of Life Sciences and Institutes of Biomedical Sciences, Fudan University, Shanghai, China; 2 Longevity Research Institute of Rugao, Jiangsu, China; 3 Department of Cellular and Structural Biology, University of Texas Health Science Center at San Antonio, San Antonio, Texas, United States of America; 4 School of Public Health, Fudan University, Shanghai, China; Innsbruck Medical University, Austria

## Abstract

**Background:**

Longevity is a multifactorial trait with a genetic contribution, and mitochondrial DNA (mtDNA) polymorphisms were found to be involved in the phenomenon of longevity.

**Methodology/Principal Findings:**

To explore the effects of mtDNA haplogroups on the prevalence of extreme longevity (EL), a population based case-control study was conducted in Rugao – a prefecture city in Jiangsu, China. Case subjects include 463 individuals aged ≥95 yr (EL group). Control subjects include 926 individuals aged 60–69 years (elderly group) and 463 individuals aged 40–49 years (middle-aged group) randomly recruited from Rugao. We observed significant reduction of M9 haplogroups in longevity subjects (0.2%) when compared with both elderly subjects (2.2%) and middle-aged subjects (1.7%). Linear-by-linear association test revealed a significant decreasing trend of N9 frequency from middle-aged subjects (8.6%), elderly subjects (7.2%) and longevity subjects (4.8%) (p = 0.018). In subsequent analysis stratified by gender, linear-by-linear association test revealed a significant increasing trend of D4 frequency from middle-aged subjects (15.8%), elderly subjects (16.4%) and longevity subjects (21.7%) in females (p = 0.025). Conversely, a significant decreasing trend of B4a frequency was observed from middle-aged subjects (4.2%), elderly subjects (3.8%) and longevity subjects (1.7%) in females (p = 0.045).

**Conclusions:**

Our observations support the association of mitochondrial DNA haplogroups with exceptional longevity in a Chinese population.

## Introduction

Individuals vary widely in their health status over their life course and in age at death. Longevity is thought to be a complex a puzzle of interplay of environmental, behavioral, genetic and stochastic factors. There is increasing evidence that exceptional longevity is a familial trait which is at least partially genetic. However, the magnitude of the genetic contribution is still controversial [1]–[3], and the inherited biological factors that promote longevity in humans remain undetermined.

Mitochondrial DNA (mtDNA) is a maternally inherited genome [4]. Human populations carry several mtDNA haplogroups defined by unique sets of mtDNA polymorphisms, reflecting mutations accumulated by a discrete maternal lineage [5], [6]. Thus, haplogroup association studies have been used to assess the effect of mtDNA variants on various complex diseases such as diabetes, cancer, and Alzheimer's disease [7]–[9]. Decades of evolution of the mitochondrial theory of aging have not yet clarified the mechanism connecting aging and accumulation of mtDNA mutations [10]. However, several aging-associated accumulations of mtDNA mutations have been reported [2], [3]. In particular, haplogroup D4a, D5, and D4b2b were observed to be enriched in centenarians in Japanese [11], [12]. Haplogroup J was detected overrepresented in male long-living subjects and centenarians in northern Italy [13], an observation that was replicated in Irish and Finnish population [14], [15], although contradicted in southern Italian [16]. These data suggest that the association between mtDNA variants and longevity could be highly geographically or population dependent, as could be seen from other genetic studies on longevity [17].

To assess the possible contribution of mtDNA haplogroup-specific mutations to the prevalence of longevity, we therefore performed a population-based case-control study in a Chinese Han population residing in Rugao, Jiangsu Province. It is worth notion that Rugao has long been claimed as a ‘longevity town’ in China since ancient time.

## Materials and Methods

### Subjects

According to the investigation of the fifth national census of China conducted in 2001, the population of Rugao was 1,362.5 thousand, and the life expectancy of Rugao is 75.58 years [18]. Until Dec, 2007, the registered number of individuals with exceptional longevity (EL, survival in good health to aged ≥95 yr) in the Longevity Institute of Rugao is 755, including 112 centenarians. The field investigation of EL subjects were conducted between Dec 24, 2007 and Feb 29, 2008. We verified the age of EL subjects by the following steps: (1) verification of dates of birth (DOB) – linkage to the 2000 population Census Office of Rugao (a part of the fifth national censuses of China) and statistic annual of Rugao; (2) verification of dates of death – linkage to the death record of local police station and Civil Administration Bureau; (3) internal consistency checks of dates by using generation of the family and the relation of the year of one's birth to one of The Twelve Animal Years which is a kind of Chinese zodiac; (4) internal consistency checks of dates in the fieldtrip. After verification, the number of persons aged ≥95 years is 705 (149 male, and 556 female), including 102 centenarians (18 male and 84 female) in Rugao. The people who had already deceased but did not deleted from the register list were excluded. Among the EL subjects, 463 subjects (360 women and 103 men; mean [SD] age, 101.23 [1.37] years; range, 95–107 years) were recruited in this study, with a response rate of 71.6%. About 94% of the EL subjects living with their offspring of three or four generations. The gender ratio of unresponded subjects did not show significant difference from that of the responded subjects (p = 0.651).

Overall 926 subjects aged 60–69 years (elderly group) were randomly recruited from the resident registry at the local government offices of Rugao. For both genders of the case subjects, two-fold elderly subjects were chosen. To test the consistency of original case-control observations, another cohort of middle-aged control subjects including 463 individuals aged 40–49 years (middle-aged group) was randomly recruited from the resident registry at the local government offices of Rugao, matched for gender with the extreme longevity group.

### Protocol and Measurements

All subjects were contacted at home and examined by physicians previously trained to administer a structured questionnaire. The questionnaire include socioeconomic status, demographic characteristics, personal habits, dietary habits, family disease histories, personal disease histories, surgery histories, menstrual and reproductive histories, hormone use histories (for women only), daily activity histories, and cognitive function appraisals, etc. Physical examinations were accomplished and fasting blood specimens were drawn for laboratory examination (e.g. lipid, glucose, liver function). For the purpose of the quality control of the investigation, all the field staff involved in data collection and data handling was trained. All interviews were tape recorded, and 5 percent of the recorded interviews were evaluated for interviewing quality. About 3–5 percent of subjects were re-contacted by phone to evaluate the interviewers' work. Written informed consent was obtained from each participant or a member of his/her immediate family. The Human Ethnics Committee of Fudan University School of Life Sciences approved the research.

### Experimental procedures

Genomic DNA was extracted by using a method described by Boom et al, with minor modifications [19]. In order to examined the correlations between the haplogroups and longevity, we selected 33 mtSNPs to define 28 major mitochondrial haplogroups (D^*^, D4, D4a, D4b, D4b2, D4b2b, D5, M12,G^*^, G2, M7^*^, M7b, M8^*^, M8a, C, M9, M10, N9, N9a, A, F^*^, F1, B^*^, B5, B5a, B5b, B4a and B4b) found in the Chinese population, as shown in Table 1 [20]. These SNPs allow allocating common mitochondrial East Asian haplotypes and following the suggestion of association of 5178A with longevity from the previous studies [11], [12], [21]–[23].

**Table 1:** Decision mtSNPs for haplogroup assignment.

Haplogroup	Key multiplex SNP variants
M*	10400T
D*	5178A 10400T
D4	3010A 5178A 10400T
D4a	14979C 3010A 5178A 10400T
D4b	8020A 3010A 5178A 10400T
D4b2	8964T 8020A 3010A 5178A 10400T
D4b2b	9296T 8964T 8020A 3010A 5178A 10400T
D5	1107C 5178A 10400T
M12	14569A 10400T
G*	4833G 14569A 10400T
G2	7600A 4833G 14569A 10400T
M7*	9824C 10400T
M7b	12811C 9824C 10400T
M8*	7196A 10400T
M8a	8684T 7196A 10400T
C	13263G 7196A 10400T
M9	4491A 3705G 10400T
M10	10646A 10400T
N*	10873T
N9*	5417A 10873T
N9a	12358G 5417A 10873T
A	1736G 10873T
F*	3970T 12705C 10873T
F1	12406A 3970T 12705C 10873T
B*	9bp- deletion 10873T
B5	8584A 9bp- deletion 10873T
B5a	15235G 8584A 9bp-deletion 10873T
B5b	15662G 8584A 9bp- deletion 10873T
B4a	5465C 9bp- deletion 10873T
B4b	13590A 9bp- deletion 10873T

We introduced the SNaPshot (Applied Biosystems, Foster City, CA) minisequencing reaction assay to genotype mitochondrial deoxyribonucleic acid single nucleotide polymorphism (SNPs) [24] with minor modifications. We genotyped the aforementioned 32 mtDNA SNPs in two independent multiplex amplifications and one SNaPshot reaction. Briefly, the PCR multiplex reaction was carried out in a total volume of 15 µL using 1.5 µL of 10X FastStart Taq DNA Polymerase PCR Buffer(Roche Diagnostics, Mannheim, Germany), 8 ng of DNA template, 2.25 µL of premixed dNTPs(Promega, Shanghai, China), 0.9 µL of 25 mM MgCl_2_ (Qiagen, Hilden, Germany), 0.12 µL of FastStart Taq DNA Polymerase(Roche Diagnostics, Mannheim, Germany) and 5.12 µL and 2.6 µL premixed primers for each multiplex amplification with the multiplex PCR cycling conditions: 95°C*4 min - [94°C*30 sec - 60°C (-0.5°C/cycle)*50 sec - 72°C*90 sec]*11cycles –[94°C*30 sec - 55°C*50 sec - 72°C*90 sec]* 24cycles - 72°C*10 min - 4°C*∞, and the final concentrations of each forward and reverse primers for mtDNA SNPs are as follows: for Multiplex 1, 12705 and 12811 are 0.33 µM; 12406 and 12358 are 0.35 µM; 7600 and 10400 are 0.53 µM; 8281 is 0.6 µM; 14569 is 0.67 µM; 9296 is 0.7 µM. For Multiplex 2, 4491, 8020, 8584 and 8684 are 0.2 µM; 5417 and 5465 are 0.25 µM; 1107 is 0.27 µM; 3010, 3970, 4833, 5178, 7196, 9824, 10646, 10873, and 15662 are 0.3 µM; 15235 is 0.37 µM; 3705, 8964, 13263 and 13590 are 0.4 µM; 14979 is 0.47 µM; 1736 is 0.5 µM. After PCR amplification, 0.95 µL of the both two multiplexes amplification products are mixed and incubated with 0.1 µL Exonuclease I (New England Biolabs, Ipswich, MA) and 2 µL shrimp alkaline phosphatase (SAP) (Promega, Madison, WI) for 80 min at 37°C followed by 15 min at 75°C for enzyme inactivation. And then, multiplex primer extension reactions were carried out by SNaPshot Multiplex Ready eaction mix (Applied Biosystems, Foster City, CA), Sequencing Buffer 5 X (Applied Biosystems, Foster City, CA) and primers extension mix with the multiplex primer extension cycling conditions: 96°C*1 min - [96°C*10 sec - 52°C*5 sec-60°C*30 sec] *28cycles - 4°C*∞ and the final concentrations of each extension primers are also given: 1107, 1736, 3010, 3705, 3970, 4491, 4833, 5178, 7196, 7600, 8020, 8584, 8684, 8964, 9824, 10646, 10873, 12811, 13263, 13590, 15235 and 15662 are 0.16 µM; 5417 is 0.33 µM; 12406 is 0.37 µM; 5465, 12358, 12705 and 14979 are 0.41 µM; 14569 is 0.45 µM; 8281, 9296 and 10400 are 0.49 µM. After the SNaPshot reaction, 6.1 µl of the SNaPshot reaction was treated with 1 U of shrimp alkaline phosphatase (Promega, Madison, WI) to inactivate the excess ddNTPs. Electrophoresis of final minisequencing products was accomplished on the ABI 3130xl Genetic Analyzer (Applied Biosystems). Genemapper ver.4.0 software was used to analyze the SNaPshot data.

### Statistical Analysis

Mitochondrial DNA haplogroup frequencies were calculated by counting from the observed genotypes. The Pearson chi-square test and the chi-square test for linear-by-linear association were used to analyze the significances between the prevalence of haplogroups and different age groups. SPSS software (version 10.0) was used.

## Results

We typed 33 mtSNPs which defined 28 haplogroups in the exceptional longevity group, elderly group, and middle-aged group. The genotyping success rates ranged from 99.5% to 100%. The prevalence of the aforementioned 28 haplogroups ranged from 1.9% to 23% in the elderly control subjects (Table 2).

**Table 2:** Distribution of mtDNA haplogroups in different age groups.

Haplogroup	Longevity	Elderly	Middle-aged	P(L vs. E)	p(L vs. M)	p-trend
D	120(25.9%)	213(23.0%)	111(24.0%)	0.23	0.494	0.387
D4	88(19.0%)	141(15.2%)	74(16.0%)	0.074	0.226	0.141
D4a	14(3.0%)	24(2.6%)	13(2.8%)	0.642	0.845	0.781
D4b	20(4.3%)	33(3.6%)	17(3.7%)	0.488	0.615	0.549
D4b2	20(4.3%)	30(3.2%)	15(3.2%)	0.308	0.389	0.319
D4b2b	14(3.0%)	25(2.7%)	11(2.4%)	0.730	0.543	0.550
D5	30(6.5%)	69(7.5%)	34(7.3%)	0.507	0.604	0.559
M12	22(4.8%)	44(4.8%)	24(5.2%)	1.000	0.762	0.796
G	21(4.5%)	39(4.2%)	21(4.5%)	0.779	1.000	0.949
G2	13(2.8%)	18(1.9%)	7(1.5%)	0.304	0.175	0.153
M7	35(7.6%)	67(7.2%)	36(7.8%)	0.827	0.902	0.955
M7b	18(3.9%)	35(3.8%)	22(4.8%)	0.921	0.518	0.587
M8	47(10.2%)	112(12.1%)	42(9.1%)	0.283	0.577	0.847
M8a	10(2.2%)	37(4.0%)	12(2.6%)	0.074	0.666	0.457
C	22(4.8%)	43(4.6%)	17(3.7%)	0.928	0.413	0.485
M9	**1(0.2%)**	**20(2.2%)**	**8(1.7%)**	0.005	0.019	0.027
M10	9(1.9%)	15(1.6%)	8(1.7%)	0.662	0.807	0.753
N9	**22(4.8%)**	**67(7.2%)**	**40(8.6%)**	0.075	0.018	0.018
N9a	18(3.9%)	51(5.5%)	31(6.7%)	0.190	0.056	0.058
A	35(7.6%)	66(7.1%)	26(5.6%)	0.770	0.233	0.288
F	77(16.6%)	138(14.9%)	61(13.2%)	0.401	0.140	0.147
F1	45(9.7%)	81(8.7%)	36(7.8%)	0.552	0.295	0.304
B	77(16.8%)	152(16.5%)	91(19.7%)	0.898	0.251	0.311
B5	29(6.3%)	39(4.2%)	20(4.3%)	0.097	0.186	0.117
B5a	16(3.5%)	22(2.4%)	8(1.7%)	0.245	0.098	0.086
B5b	13(2.8%)	16(1.7%)	12(2.6%)	0.186	0.839	0.357
B4a	9(1.9%)	31(3.4%)	17(3.7%)	0.139	0.112	0.105
B4b	8(1.7%)	24(2.6%)	11(2.4%)	0.310	0.487	0.430

L: longevity group.

E: elderly group.

M: Middle-aged group.

Significant reduction of M9 haplogroups was observed in EL subjects (0.2%) when compared with both elderly subjects (2.2%) and middle-aged subjects (1.7%) (p = 0.005 and p = 0.019, respectively). Linear-by-linear association test revealed a significant decreasing trend of N9 frequency from middle-aged subjects (8.6%), elderly subjects (7.2%) and EL subjects (4.8%) (p = 0.018) Table 2.

In subsequent analysis stratified by gender, linear-by-linear association test revealed a significant increasing trend of D4 frequency from middle-aged subjects (15.8%), elderly subjects (16.4%) and EL subjects (21.7%) in females (p = 0.025) (Table 3). Conversely, a significant decreasing trend of B4a frequency was observed from middle-aged subjects (4.2%), elderly subjects (3.8%) and longevity subjects (1.7%) in females (p = 0.045). However, although a decreasing trend of N9 haplogroups was still observed from middle-aged subjects (8.6%), elderly (7.1%) and EL subjects (5.3%) in female samples, the statistical significance originally observed in all subjects diminished, which may be due to lack of statistic power (Table 3).

**Table 3:** Distribution of mtDNA haplogroups in female subjects of different age groups.

Haplogroups	Longevity	Elderly	Middle-aged	p(L vs. E)	p(L vs. M)	p-trend
D4	78(21.7%)	118(16.4%)	57(15.8%)	0.034	0.045	0.025
B4a	6(1.7%)	27(3.8%)	15(4.2%)	0.060	0.046	0.045
M9	0(0%)	14(1.9%)	6(1.7%)	0.008	0.014	0.032
N9	19(5.3%)	51(7.1%)	31(8.6%)	0.256	0.079	0.082

## Discussion

In the present study, we conducted a population-based case-control study to explore the effects of mtDNA haplogroups on the phenomenon of extremely long life in a Chinese population. Crucial to our approach is the strong survival advantage of the extremely longevity people at Rugao (less than 1/10,000 Rugao people survive to 100 y, while the average lifespan in Rugao is 75.58 y). The enrichment in the frequency of certain genetic variants at this extremely old people probably reflects a selection effect that may enhance the likelihood of survival. We did observe the enrichment of certain haplogroups in Rugao population, such as D4 haplogroup. Interestingly, there is a reduction of M9, N9, and B4a in these long lived subjects.

Haplogroups D4 is one of the most characteristic mtDNA lineages found in Northeast Asian populations [25]. Historically, the population of Rugao was mainly admixed with the local residents and the immigrants from northern China in the last two millenniums [26]. It was reported that the 5178A of haplogroup D4 was enriched in Japanese centenarians [21]. Haplogroups D4a, D5, and D4b2 were significantly enriched in male centenarians compared with healthy young Japanese males. [12]. Further, D4a haplogroup were associated with certain beneficial patterns in Japanese centenarians and semi-supercentenarians [11]. In the present study, we also observed enrichment of D4a haplogroup in Rugao longevity subjects than two control groups, albeit significance only exists in females. Our results provided evidence that D4a contribute to longevity outside Japan.

Haplogroups M9 and N9, two major subclusters directly sprout from the basal M and N trunks, respectively, have originated from Southeast Asia conceivably and are prevailing over central or northern China [27]–[29]. Significantly higher prevalence of M9 was observed in Tibetan (20%) than in Chinese Han (2.0%) living in northern China. In Rugao subjects living in an area where admixture of southern and northern populations occurred, the prevalence of M9 (2.2%) is similar to those living in northern China. The specific characteristic mutation to M9 is G4491A from valine (V) to isoleucine (I), which is considered a potentially functional polymorphism. Nevertheless, although the presence of M9 haplogroup is negatively associated with the risk of longevity in Rugao population, the prevalence of M9 is only 2.2%. Therefore, the attributable risk of M9 to the longevity is not prominent at Rugao. In the present study, we presented the first report that N9 confers an increased risk against longevity. The specific characteristic mutation 5417 to N9 had silent amino acid change [30], [31]. Thus, the potential mechanism for the involvement of N9 in longevity in female of Rugao calls for further investigation. Interestingly, it was reported recently that haplogroup N9a confers resistance against type 2 diabetes in Asians and haplogroup N9a protected against metabolic syndrome in Japanese women [32], [33]. These may relate to N9a-specific mtSNPs or other potentially functional polymorphisms. The B4a haplogroups, defined as a subgroup of B4 by the presence of the 16261T polymorphism, are frequent along the southeastern coast of Taiwan, but also have a wide and ancient distribution all over East Asia and island Pacific [34], [35]. To the best of our knowledge, there is no report linking B4a to the phenotype of complex trait. Therefore, our observation that B4a is negatively correlated with ages in Rugao population has never been reported elsewhere.

In genetic association studies on longevity, the gender difference is common, where different polymorphisms of mtDNA were associated with longevity only in males [12], [14], [15]. The reason for such a gender difference is not clear. A possible reason for the observed gender difference could be that males are under stronger selection than females, and are thus less likely to reach the same age as women [36]. The male-specific association between mtDNA haplogroups and longevity is consistent with the hypothesis that mtDNA variations have a higher impact on male than on female longevity [37]. This gender difference could be one of the consequences of the maternal inheritance of mtDNA, which in turn implies a higher relaxation and less purging of male-specific mitochondrial phenotype, being males' evolutionary dead ends for mtDNA. Interestingly, the haplogroup D4 was not enriched in male Rugao subjects, but only in female subjects. Coincidently, longevity also shows maternal inheritance [38], and the female male ratio is 3.5∶1 in Rugao long lived subjects. The fundamental mechanism of this phenomenon and the female correlation warrants further exploring.

Recently, haplogroup association studies have been used to define the role of mtDNA mutations in complex diseases and longevity. In this approach, case and control mtDNA sequence sets are assigned to haplogroups, and the distribution of haplogroups is compared. A significant difference is interpreted to indicate that the mtDNA ‘background’ has an effect on expression or penetrance of the phenotypes. Given the inconsistency results among the different studies, as observed in Rugao population and Japanese [11], [37]; it is difficult to conclude unequivocally that longevity is influenced by mitochondrial haplogroups. The nuclear background in which these mtDNA haplotypes are found is a factor that may contribute to difficulty in detecting and interpreting correlations between mitochondrial haplogroups and longevity. As early as 1996, Guan et al [39] provided biochemical evidence for nuclear gene involvement in phenotype of non-syndromic deafness associated with mitochondrial 12S rRNA mutation. However, inconsistency may also be due to population-substructure, lacking of power for small sample size, and possibly different genomic background. Firstly, it is difficult to ‘match’ groups, even when one tries to control gender, ethnicity, and geography. Secondly, the recruitment of cases and control groups from separate population groups is another common source of bias in case-control studies. However, in this study, all cases were recruited from the Han ethnicity of the Rugao City, with a high response rate. The gender ratio of unresponded subjects is similar to the responded ones. Therefore, the representative of the case subjects to source population is guaranteed. The sample frame of our control group was also exerted from the original population of case group and well matched for gender with the case group, thus minimized the population stratification and false positive results. The population was relatively homogeneous related to environmental exposures, which decreased the selection biases for case control matching. Finally, although our study included 463 cases and two control groups, unfortunately, interpreting the results is also limited by the relatively insufficient sample size. For example, our sample size had the power of 0.91, and 0.65 to detect the differences between longevity subjects and elderly subjects, between longevity subjects and middle-aged subjects, respectively, given the significant level of 0.05. However, when applying a conservative Bonferroni correction for multiple testing, the significant level will be set at 0.05/28 for 28 haplogroups we analyzed. Nevertheless, for D super-haplogroup, our aim was to validate the observations made by Bilal et al and Alexe et al [11], [12], we still set the significant level at α = 0.05.

In summary, we observed the involvement of mtDNA haplogroups in the prevalence of longevity by using a carefully matched population based case-control design in Rugao, China. The results should lead to increasing understanding of the role of genome maintenance in longevity. Although we selected two control groups to verify our discovery, our finding also needs to be replicated by other independent studies.
